# Quality of Hospices Used by Medicare Advantage and Traditional Fee-for-Service Beneficiaries

**DOI:** 10.1001/jamanetworkopen.2024.51227

**Published:** 2024-12-16

**Authors:** Lindsay L. Y. White, Chuxuan Sun, Norma B. Coe

**Affiliations:** 1 Department of Medical Ethics and Health Policy, Perelman School of Medicine, University of Pennsylvania, Philadelphia

## Abstract

**Importance:**

Although enrollment in both hospice care and Medicare Advantage (MA) have grown substantially, little is known about the quality of hospice care received by MA beneficiaries relative to traditional Medicare fee-for-service (FFS) beneficiaries.

**Objective:**

To compare hospice enrollment and the quality of hospices serving MA and FFS beneficiaries.

**Design, Setting, and Participants:**

This population-based cross-sectional study used Medicare enrollment and claims data from January 1, 2018, through December 31, 2019, and Hospice Quality Reporting Program (HQRP) data released between November 1, 2020, and August 30, 2022, to compare the probability of enrolling in hospice before death and the probability of using high- vs low-quality hospices between MA and FFS beneficiaries. Two sample populations were assessed: (1) all Medicare beneficiaries who died in 2018 or 2019, and (2) all Medicare hospice enrollees in 2018 and 2019, excluding beneficiaries with hospice use in 2017. Data were analyzed between April 1, 2023, and April 30, 2024.

**Exposures:**

MA enrollment was assessed 6 months prior to death for decedents and in the month of hospice admission for hospice enrollees. MA beneficiaries were further classified by plan type: regular MA, special needs plan (SNP), and Medicare-Medicaid plan (MMP).

**Main Outcomes and Measures:**

For decedents, the outcome of interest was the prevalence of any hospice use in the last 6 months of life. For hospice enrollees, the outcome of interest was 9 HQRP measures of hospice quality.

**Results:**

Data from 4 215 648 decedents (51.6% female; mean [SD] age, 80.1 [11.6] years) and 2 211 826 hospice enrollees (56.6% female; mean [SD] age, 82.4 [10.5] years) were included. In the decedent sample, beneficiaries enrolled in every type of MA plan were significantly more likely than beneficiaries enrolled in FFS to use hospice care in the last 6 months of life (regular MA beneficiaries were 3.4 percentage points more likely to use hospice; MA SNP beneficiaries, 2.4 percentage points; and MA MMP beneficiaries, 3.6 percentage points). Regular MA and FFS beneficiaries enrolled in hospices of similar quality. However, beneficiaries in SNPs and MMPs were significantly more likely than FFS beneficiaries to use hospices with inferior quality (eg, MA SNP beneficiaries were 4.3 [95% CI, 3.9-4.7] percentage points more likely to use a hospice with a low Consumer Assessment of Healthcare Providers and Systems (CAHPS) global rating, and MA MMP beneficiaries were 6.8 [95% CI, 6.0-7.7] percentage points more likely). When beneficiaries entered hospice from the same hospital or nursing home the results were attenuated: the MA SNP beneficiaries entering from the same hospital were 0.9 (95% CI, 0.5-1.4) percentage points more likely to use a hospice with a low CAHPS global rating, and MA MMP beneficiaries were 3.8 (95% CI, 2.4-5.1) percentage points more likely; MA SNP beneficiaries entering from the same nursing home were 2.8 (95% CI, 2.3-3.3) percentage points more likely to use a hospice with a low CAHPS global rating, and MA MMP beneficiaries were 1.9 (95% CI, 0.9-2.9) percentage points more likely. These results suggest that referral networks were an important mechanism of the hospice quality choice.

**Conclusions and Relevance:**

These findings suggest that policymakers should consider policies for MA programs that incentivize referrals to high-quality hospices and approaches to educating beneficiaries on identifying high-quality hospice care.

## Key Points

**Question:**

Do beneficiaries of Medicare Advantage and traditional Medicare fee-for-service use hospices of the same quality?

**Findings:**

In this cross-sectional study including 4 215 648 decedents and 2 211 826 hospice enrollees, regular Medicare Advantage and fee-for-service beneficiaries enrolled in hospices of similar quality. However, beneficiaries in Medicare Advantage special needs plans were significantly more likely than fee-for-service beneficiaries to use hospices of inferior quality, with referral networks playing an important role in hospice quality choice.

**Meaning:**

These results suggest that policymakers should consider incentivizing referrals to high-quality hospices and approaches to educating beneficiaries on identifying high-quality hospice care.

## Teaser

This cross-sectional study compares the likelihoods of enrolling in hospice before death and of using high- vs low-quality hospice care between beneficiaries of Medicare Advantage and Medicare fee-for-service.

## Introduction

In 2023, enrollment in the Medicare Advantage (MA) program increased to 30.8 million people, or just over half of all Medicare beneficiaries.^1^ The MA plans are paid a risk-adjusted, capitated rate to provide comprehensive health services to beneficiaries. Consequently, MA plans have a strong incentive to limit the costs of care for their enrollees, and they may do so through a variety of strategies, including prioritizing preventive care,^2,3,4,5^ using utilization controls (eg, prior authorization for services),^6^ and selectively contracting with medical institutions willing to accept lower reimbursement rates.^7,8,9^ Several prior studies suggested that contracting with these lower-cost institutions may come at the cost of care quality; compared with beneficiaries in the traditional Medicare fee-for-service (FFS) program, beneficiaries in MA are more likely to receive services from lower-quality hospitals, home health agencies, and skilled nursing facilities.^10,11,12^

Hospice use has increased markedly during the past 2 decades, with slightly fewer than half of recent Medicare decedents receiving hospice care at the time of death (47.2% and 47.4% of FFS and MA decedents, respectively, in 2022).^13^ Hospice services are unique in that they are not included in the MA benefits package but are instead paid through FFS. When an MA enrollee elects hospice, they can retain their MA coverage or move entirely to FFS. If they retain their MA coverage, the MA plan is paid at a reduced rate and is responsible for services unrelated to the terminal condition (eg, treatment and rehabilitation services after a traumatic injury) as well as supplemental benefits; all services related to the beneficiary’s terminal condition are paid through FFS.

Given their lack of hospice benefits, MA plans have different financial incentives around the timing of hospice care initiation and the hospice agency to which enrollees are referred than they do with other covered services. For example, MA plans have a financial incentive to refer beneficiaries to hospice if they are nearing the end of life and are using high intensity services, as it removes costly beneficiaries from the plan rolls. Studies demonstrate historically higher rates of hospice enrollment among decedents in MA compared with those in FFS,^14^ although these trends have recently equalized.^13^ In selecting a hospice to refer beneficiaries to, MA plans are not allowed to restrict to preferred agency networks and do not have an incentive to refer to lower-cost agencies, as they have with other services. Thus, prior work on the quality of nonhospice agencies serving MA beneficiaries may not generalize to hospice care.

Little is known about how the quality of hospice care received by MA beneficiaries compares with that received by FFS beneficiaries. One prior study demonstrated differential care quality between MA and FFS hospice users, with friends and family of MA decedents more likely to report poorer quality of hospice care.^15^ While explicit hospice agency networks are not permitted within MA plans, limited networks for other services may create implicit hospice networks through agency referrals. For example, a hospital stay is the most common entry point to hospice care for Medicare beneficiaries.^16^ By receiving services through a limited hospital network, MA beneficiaries may be steered toward certain hospices favored by the hospitals.

In this study, we used Medicare enrollment and claims data along with data from the Hospice Quality Reporting Program (HQRP) to evaluate both differences in hospice enrollment and the quality of the hospices that served MA and FFS beneficiaries in 2018 and 2019. Since 1 in 5 MA beneficiaries is enrolled in a special needs plan (SNP),^1^ plans specifically designed to provide coordinated care for populations with unique and heightened health care needs, including dual-eligible SNPs (D-SNPs), chronic condition SNPs (C-SNPs), institutional SNPs (I-SNPs), and Medicare-Medicaid Plans (MMPs), we stratified MA beneficiaries by plan type.

## Methods

### Study Design and Population

This study was approved by the University of Pennsylvania Institutional Review Board, which granted a waiver of the requirement of obtaining informed consent because of the infeasibility of obtaining consent and the minimal risk of harm. Reporting followed the Strengthening the Reporting of Observational Studies in Epidemiology (STROBE) reporting guideline for cross-sectional studies. Data were analyzed between April 1, 2023, and April 30, 2024.

We conducted a cross-sectional study of the association between MA enrollment and both hospice enrollment and hospice care quality. We identified all Medicare beneficiaries who died January 1, 2018, through December 31, 2019, from the Master Beneficiary Summary file (MBSF), and all hospice admissions from January 1, 2018, through December 31, 2019, using the hospice claims (population flowchart in the eFigure in Supplement 1). Since the choice of hospice is likely influenced by any prior hospice experience, we excluded all but the first hospice admission for beneficiaries with multiple stays, and we excluded beneficiaries with any hospice use in 2017 from our hospice enrollee cohort. We also excluded decedents and hospice enrollees who were missing MA plan information. Finally, the choice to enroll in hospice and the specific hospice selected is likely influenced by geographic access. To account for this, we compared MA and FFS enrollees living within the same zip code, and consequently excluded beneficiaries residing in zip codes with fewer than 10 MA and 10 FFS decedents for our decedent analyses, or fewer than 10 MA and 10 FFS hospice enrollees for our hospice quality analyses, to minimize the effect of zip codes with limited MA or FFS penetration.

### Outcome Variables

For our decedent cohort, our outcome of interest was any hospice use in the last 6 months of life, which was determined using hospice claims. For our hospice enrollee cohort, hospice quality was derived from HQRP data, which includes data submitted by hospices, data from hospice claims, and survey responses from the Consumer Assessment of Healthcare Providers and Systems (CAHPS) Hospice Survey. Participation in the HQRP is mandatory for Medicare-certified hospices, although there are reporting exemptions for low patient volume and recent Medicare certification.

We used 8 current HQRP measures and 1 additional measure that was in use during our study period (eTable 1 in Supplement 1). Specifically, we used the CAHPS global hospice rating measure from the November 2020 release, which reflects care provided in 2018 and 2019. We also used 2 hospice-reported measures from the May 2021 release: Hospice Item Set Comprehensive Assessment at Admission, and Hospice Visits When Death is Imminent, corresponding to care provided in 2019. Finally, we used 5 claims-based measures—Hospice Visits in Last Days of Life and 4 components of the Hospice Care Index (components not used exhibited very little variation in scores as did the composite measure) as well as the CAHPS Hospice Survey Star Rating from the August 2022 release—that measured care provided from April 1, 2019, through December 31, 2019, and from July 1, 2020, through September 30, 2021.

For the hospice-reported and claims-based measures, we used quintiles of performance^12^ (eTable 2 in Supplement 1 gives the distribution of each measure and the specific cut points used), and defined hospices in the highest quintile as having high quality vs those in the lowest quintile as having low quality. For the CAHPS global rating measure, we categorized agencies with scores of 3 or more points above or below the national hospice average as high- and low-quality agencies, respectively.^17^ Finally, hospices with a CAHPS Hospice Survey Star Rating of 4 or 5 were considered high quality, and agencies with 1 or 2 stars were considered low quality.

### Explanatory Variables

Our explanatory variables, MA enrollment 6 months prior to death for our decedent cohort and in the month of hospice admission for our hospice enrollee cohort, were determined using the MBSF monthly Medicare Part C indicators. We stratified MA enrollees on MA plan type (regular MA, SNP [including D-SNP, C-SNP, and I-SNP], and MMP) using data from the Landscape Source files and the monthly MA contract and enrollment files. We considered beneficiaries in MMPs separately from other SNP beneficiaries because MMPs offer full integration of benefits and services across Medicare and Medicaid.

### Covariates

The CAHPS measures are adjusted for case mix prior to their public release. Consequently, we controlled only for sex, race and ethnicity as determined using the Research Triangle Institute race variable in the MBSF (African American or Black, Hispanic, non-Hispanic White, and other race or ethnicity [Asian or Pacific Islander, American Indian or Alaska Native, and other]), and the original reason for Medicare entitlement in our analyses with CAHPS measures. Race and ethnicity were based on data from the Social Security Administration, to which an algorithm is applied to improve classification of people of Hispanic and Asian origin. For all other analyses, we also included age and dual eligibility with Medicaid as covariates. Within our hospice enrollee analyses, we also controlled for principal hospice diagnosis (cancer, congestive heart failure, chronic obstructive pulmonary disease, cerebrovascular accident, Alzheimer disease and related dementias, end-stage kidney disease, and all other diagnoses).^18^

### Statistical Analysis

We fit linear probability models with zip code fixed effects and zip code clustered standard errors within separate MA plan type strata to estimate the association between MA plan enrollment and our outcomes, while accounting for both beneficiary characteristics and geographic access. To explore the role of hospice referral source, we determined place of care prior to hospice enrollment using Medicare Provider Analysis and Review and the Minimum Data Set 3.0 files for January 1, 2017, through December 31, 2019. Beneficiaries receiving care at a hospital or nursing home within 7 days of hospice admission were classified as entering from the institution. We then fit separate linear probability models with hospital and nursing home fixed effects (with hospital or nursing home clustered standard errors) to allow for a comparison of MA and FFS beneficiaries entering hospice from the same institution.

We conducted several sensitivity analyses. First, since MA plan quality may be associated with agency quality,^10,11,12^ we further classified MA enrollees by plan quality. Enrollees in 4.0 to 5.0 star-rated plans were classified as having higher-quality MA, while enrollees in 1.0 to 3.5 star-rated plans were classified as having lower-quality MA. Second, because the 3 types of SNPs may have differing incentives to enroll beneficiaries in hospice, we split the SNP group by SNP type. Third, to assess concerns that our zip code requirements were too restrictive, we re-ran our models with county fixed effects in place of zip code fixed effects (requiring a minimum of 10 MA and 10 FFS decedents or hospice enrollees within the county rather than zip code). Fourth, for our hospice quality models, we further limited our sample to beneficiaries residing in zip codes that had at least 1 high-quality hospice serving the zip code during our study period (high quality was specific to the outcome measure used in the model) to ensure the availability of high-quality options. Finally, we excluded beneficiaries under 65 years of age from analyses because end-of-life care patterns may differ between older and younger adults. All analyses were conducted in Stata, version 17 (StataCorp LLC). Statistical significance was defined as a 95% CI excluding 0.

## Results

### Decedents

Our decedent sample included 4 215 648 beneficiaries (51.6% female and 48.4% male; 10.7% African American or Black, 6.5% Hispanic, 78.8% non-Hispanic White, and 4.0% unknown or other race and ethnicity); mean [SD] age, 80.1 [11.6] years). Over one-third (35.0%) were enrolled in MA in the 6 months prior to death (Table 1), 82.8% in a regular MA plan, 14.7% in an SNP, and 2.5% in an MMP. Compared with both FFS and regular MA beneficiaries, enrollees in an MA SNP or MMP were less likely to be non-Hispanic White (80.5% in FFS, 80.1% in regular MA, 54.4% in MA SNP, and 49.2% in MA MMP) and more likely to be dually enrolled in Medicaid (27.5% in FFS, 14.0% in regular MA, 86.2% in MA SNP, and 99.4% in MA MMP). In unadjusted analyses, regular MA beneficiaries were more likely than FFS beneficiaries to have any hospice use in the last 6 months of life (55.2% vs 50.4%), while beneficiaries in an SNP were less likely (47.8%). In our multivariable regression model (Figure), MA decedents in all plan types had a significantly higher probability than FFS beneficiaries of using any hospice care in the 6 months before death (regular MA beneficiaries were 3.4 percentage points more likely to use hospice; MA SNP beneficiaries, 2.4 percentage points; and MA MMP beneficiaries, 3.6 percentage points).

**Table 1.** Characteristics of Decedents by Insurance Type

Characteristic	Decedents, No. (%) with fee-for-service Medicare	Decedents, No. (%) with Medicare Advantage (n = 1 474 275)		
		Regular Medicare Advantage	Special needs plan	Medicare-Medicaid Plan
Beneficiaries, No.	2 741 373	1 221 155	216 047	37 073
Age, mean (SD), years	80.0 (12.0)	81.1 (10.2)	77.4 (12.7)	77.2 (14.0)
Sex				
Female	1 408 842 (51.4)	615 887 (50.4)	126 684 (58.6)	22 211 (59.9)
Male	1 332 531 (48.6)	605 268 (49.6)	89 363 (41.4)	14 862 (40.1)
Race and ethnicity				
African American or Black	273 342 (10.0)	120 724 (9.9)	48 922 (22.6)	8827 (23.8)
Hispanic	149 149 (5.4)	80 510 (6.6)	36 530 (16.9)	7236 (19.5)
Non-Hispanic White	2 208 024 (80.5)	978 307 (80.1)	117 608 (54.4)	18 251 (49.2)
Other^a^	92 436 (3.4)	35 804 (2.9)	12 018 (5.6)	2551 (6.9)
Unknown	18 422 (0.7)	5810 (0.5)	969 (0.5)	208 (0.6)
Dual enrollment 6 mo prior to death				
Nondual	1 987 725 (72.5)	1 049 474 (85.9)	29 711 (13.8)	217 (0.6)
Partial dual	115 527 (4.2)	63 868 (5.2)	36 101 (16.7)	88 (0.2)
Full dual	638 121 (23.3)	107 813 (8.8)	150 235 (69.5)	36 768 (99.2)
Original reason for Medicare entitlement–Disability or ESKD	601 007 (21.9)	221 900 (18.2)	78 010 (36.1)	13 219 (35.7)
Hospice use in the last 6 mo of life	1 381 753 (50.4)	673 871 (55.2)	103 177 (47.8)	18 409 (49.7)

Abbreviation: ESKD, end-stage kidney disease.

^a^ Other race and ethnicity included the following categories from the Research Triangle Institute race variable: Asian or Pacific Islander, American Indian or Alaska Native, and other.

**Figure.**
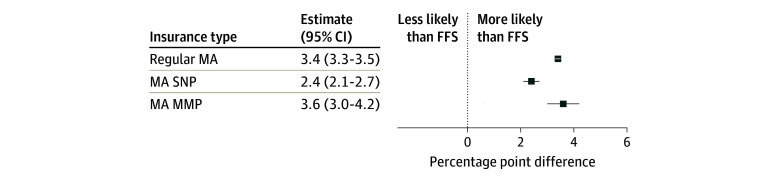
Percentage Point Difference in the Probability of Hospice Use in the Last 6 Months of Life FFS indicates fee-for-service; MA, Medicare Advantage; MMP, Medicare-Medicaid Plan; and SNP, special needs plan.

### Hospice Enrollees

Our hospice enrollee sample included 2 211 826 beneficiaries (56.6% female and 43.4% male; 8.6% African American or Black, 6.3% Hispanic, 81.9% non-Hispanic White, and 3.3% unknown or other race and ethnicity; mean [SD] age, 82.4 [10.5] years). Of them, 38% were enrolled in MA at hospice admission (83.4% in regular MA, 14.2% in MA SNP, and 2.4% in MA MMP) (eTable 3 in Supplement 1). All MA beneficiaries were less likely than FFS beneficiaries to have a hospitalization within 7 days of hospice enrollment (44.5% in FFS, 39.3% in regular MA, 38.7% in MA SNP, and 37.9% in MA MMP). Patterns were more mixed on prevalence of having a nursing home stay within 7 days of hospice admission (30.4% for FFS vs 22.2% for regular MA, 40.1% for MA SNP, and 55.0% for MA MMP). MA beneficiaries of all plan types were more likely to use a for-profit hospice than FFS beneficiaries (44.8% in FFS, 46.2% in regular MA, 51.3% MA SNP, and 61.1% in MA MMP).

In unadjusted analyses (Table 2), MA beneficiaries were more likely to use a hospice in the lowest level of quality performance and less likely to use a hospice in the highest quality level than FFS beneficiaries, irrespective of the type of their MA plan. In adjusted analyses (Table 2), differences between FFS and regular MA beneficiaries in the probability of using a high- or low-quality hospice were nonsignificant or, if significant, of very small magnitude and inconsistent in direction. However, adjusted differences consistently indicated that MA SNP and MMP beneficiaries enrolled in lower-quality hospices than FFS beneficiaries. For example, beneficiaries in an SNP were 4.3 (95% CI, 3.9-4.7) percentage points more likely than FFS beneficiaries to use a hospice with a low CAHPS global rating, while MMP beneficiaries were 6.8 (95% CI, 6.0-7.7) percentage points more likely. When controlling for the referring hospital among beneficiaries entering hospice from the hospital setting (eg, MA SNP coefficient of 0.009 [95% CI, 0.005-0.014] and MA MMP coefficient of 0.038 [95% CI, 0.024-0.051] for low quality CAHPS global rating, indicating MA SNP beneficiaries were 0.9 percentage points more likely to use a hospice with a low CAHPS global rating, and MA MMP beneficiaries were 3.8 percentage points more likely) (Table 3) or for the referring nursing home among beneficiaries entering from a nursing home (eg, MA SNP coefficient of 0.028 [95% CI, 0.023-0.033] and MA MMP coefficient of 0.019 [95% CI, 0.009-0.029] for low quality CAHPS global rating, indicating MA SNP beneficiaries were 2.8 percentage points more likely to use a hospice with a low CAHPS global rating, and MA MMP beneficiaries were 1.9 percentage points more likely) (Table 4), the results were attenuated, that is, they were smaller in magnitude and fewer comparisons were statistically significant.

**Table 2.** Association Between Insurance Type and Probability of Enrollment in Low- or High-Quality Hospices

Quality measure	Medicare Fee-for-Service, unadjusted % of enrollees	Regular Medicare Advantage		Special Needs Plan		Medicare-Medicaid Plan	
		Unadjusted, % of enrollees	Adjusted difference, % (95% CI)	Unadjusted, % of enrollees	Adjusted difference, % (95% CI)	Unadjusted, % of enrollees	Adjusted difference, % (95% CI)
HIS composite							
Low quality	8.5	8.9	0.2 (0.1 to 0.3)	8.8	−0.2 (−0.4 to 0.0)	11.9	1.6 (1.0 to 2.1)
High quality	29.1	28.2	−0.2 (−0.4 to −0.1)	25.9	−1.6 (−1.9 to −1.2)	26.9	−1.0 (−1.8 to −0.3)
Missing	1.4	1.5	NA	1.7	NA	3.0	NA
Hospice visits when death is imminent							
Low quality	16.0	18.9	0.0 (−0.1 to 0.2)	22.7	0.9 (0.6 to 1.2)	21.8	1.5 (0.9 to 2.0)
High quality	11.2	9.9	−0.2 (−0.3 to −0.1)	9.4	−0.5 (−0.7 to −0.3)	6.2	0.1 (−0.3 to 0.6)
Missing	2.5	2.9	NA	3.3	NA	6.6	NA
Hospice visits in the last days of life							
Low quality	5.7	7.6	0.1 (0.0 to 0.2)	8.6	0.6 (0.4 to 0.9)	13.6	1.8 (1.3 to 2.3)
High quality	18.5	16.4	−0.8 (−0.9 to −0.7)	15.6	−0.3 (−0.6 to −0.0)	13.7	−0.1 (−0.6 to 0.5)
Missing	2.2	2.3	NA	2.2	NA	3.2	NA
HCI: gaps in skilled nursing visits							
Low quality	19.9	19.7	−0.1 (−0.2 to −0.0)	26.3	1.1 (0.8 to 1.4)	25.9	2.7 (2.0 to 3.4)
High quality	12.4	11.5	−0.3 (−0.4 to −0.2)	9.9	−0.6 (−0.9 to −0.4)	5.2	−0.6 (−1.0 to −0.2)
Missing	2.1	2.2	NA	2.1	NA	3.1	NA
HCI: late live discharges							
Low quality	9.0	10.2	−0.3 (−0.4 to −0.2)	11.4	0.6 (0.3 to 0.9)	15.2	−0.1 (−0.7 to 0.5)
High quality	13.8	14.6	0.9 (0.8 to 1.1)	10.9	−0.0 (−0.2 to 0.2)	11.2	−0.6 (−1.1 to −0.1)
Missing	2.1	2.2	NA	2.1	NA	3.1	NA
HCI: burdensome transitions, type 1							
Low quality	13.8	11.9	−0.3 (−0.4 to −0.2)	12.3	−0.2 (−0.5 to 0.0)	17.7	2.0 (1.4 to 2.7)
High quality	8.6	11.2	1.3 (1.2 to 1.5)	9.5	0.3 (0.1 to 0.6)	4.0	−0.2 (−0.6 to 0.2)
Missing	2.1	2.2	NA	2.1	NA	3.1	NA
HCI: skilled nursing care min per routine home care day							
Low quality	13.3	11.5	−0.1 (−0.2 to −0.0)	17.2	0.8 (0.5 to 1.0)	16.0	0.0 (−0.6 to 0.7)
High quality	22.0	24.1	0.1 (−0.0 to 0.3)	20.5	−0.6 (−1.0 to −0.3)	24.7	−0.6 (−1.3 to 0.1)
Missing	2.1	2.2	NA	2.1	NA	3.1	NA
CAHPS global rating							
Low quality	29.8	31.9	0.2 (0.1 to 0.4)	39.6	4.3 (3.9 to 4.7)	41.5	6.8 (6.0 to 7.7)
High quality	30.6	27.4	−0.5 (−0.6 to −0.3)	21.8	−3.5 (−3.8 to −3.2)	20.0	−4.7 (−5.4 to −4.0)
Missing	4.9	5.6	NA	6.6	NA	10.8	NA
Hospice star rating							
Low quality	16.5	18.1	0.2 (0.0 to 0.3)	23.9	2.7 (2.3 to 3.0)	22.4	2.8 (2.0 to 3.6)
High quality	34.4	30.4	−0.3 (−0.4 to −0.1)	26.1	−2.2 (−2.5 to −1.8)	14.4	−2.5 (−3.2 to −1.9)
Missing	13.0	13.1	NA	15.2	NA	21.9	

Abbreviations: CAHPS, Consumer Assessment of Healthcare Providers and Systems; HCI, Hospice Care Index; HIS, Hospice Item Set; NA, not applicable.

**Table 3.** Probability of Enrollment in Low- or High-Quality Hospices When Beneficiaries Enter Hospice From the Same Hospital, Compared With Medicare Fee-for-Service

Quality measure^a^	Medicare Advantage, coefficient (95% CI)		
	Regular Medicare Advantage	Special Needs Plan	Medicare-Medicaid Plan
HIS composite			
Low quality	0.000 (−0.001 to 0.002)	−0.001 (−0.004 to 0.002)	0.004 (−0.003 to 0.012)
High quality	0.001 (−0.002 to 0.003)	−0.001 (−0.006 to 0.004)	0.010 (−0.003 to 0.022)
Hospice visits when death is imminent			
Low quality	0.002 (0.001 to 0.004)	0.005 (−0.000 to 0.009)	0.013 (0.003 to 0.023)
High quality	−0.002 (−0.003 to −0.001)	−0.000 (−0.004 to 0.003)	−0.007 (−0.013 to −0.002)
Hospice visits in the last days of life			
Low quality	0.001 (−0.001 to 0.002)	0.001 (−0.002 to 0.004)	0.006 (−0.002 to 0.015)
High quality	−0.004 (−0.005 to −0.002)	0.000 (−0.004 to 0.004)	0.001 (−0.007 to 0.009)
HCI: gaps in skilled nursing visits			
Low quality	−0.001 (−0.002 to 0.001)	0.002 (−0.002 to 0.006)	0.015 (0.003 to 0.027)
High quality	−0.002 (−0.004 to −0.001)	−0.005 (−0.010 to −0.000)	−0.008 (−0.016 to −0.000)
HCI: late live discharges			
Low quality	−0.000 (−0.002 to 0.001)	0.002 (−0.001 to 0.005)	0.005 (−0.004 to 0.015)
High quality	0.000 (−0.001 to 0.002)	−0.002 (−0.006 to 0.001)	0.006 (−0.004 to 0.017)
HCI: burdensome transitions to type 1			
Low quality	−0.001 (−0.002 to 0.001)	0.002 (−0.003 to 0.006)	0.003 (−0.008 to 0.014)
High quality	0.001 (−0.000 to 0.003)	−0.002 (−0.004 to 0.000)	−0.007 (−0.011 to −0.002)
HCI: skilled nursing care minutes per routine home care day			
Low quality	−0.001 (−0.003 to 0.001)	−0.001 (−0.005 to 0.002)	−0.004 (−0.013 to 0.005)
High quality	−0.002 (−0.004 to 0.000)	−0.004 (−0.010 to 0.001)	−0.002 (−0.014 to 0.010)
CAHPS global rating			
Low quality	0.004 (0.001 to 0.006)	0.009 (0.005 to 0.014)	0.038 (0.024 to 0.051)
High quality	−0.005 (−0.006 to −0.003)	−0.012 (−0.015 to −0.008)	−0.024 (−0.035 to −0.013)
Hospice star ratin**g**			
Low quality	0.001 (−0.001 to 0.002)	0.006 (0.000 to 0.012)	0.028 (0.014 to 0.042)
High quality	−0.003 (−0.005 to −0.001)	−0.008 (−0.013 to −0.003)	−0.013 (−0.024 to −0.003)

Abbreviations: CAHPS, Consumer Assessment of Healthcare Providers and Systems; HCI, Hospice Care Index; HIS, Hospice Item Set.

^a^ Beneficiaries with Medicare fee-for-service is the reference for these comparisons.

**Table 4.** Probability of Enrollment in Low- or High-Quality Hospices When Beneficiaries Enter Hospice From the Same Nursing Home, Compared With Medicare Fee-for-Service

Quality measure^a^	Medicare Advantage, coefficient (95% CI)		
	Regular Medicare Advantage	Special Needs Plan	Medicare-Medicaid Plan
HIS composite			
Low quality	−0.000 (−0.002 to 0.002)	0.002 (−0.002 to 0.005)	0.005 (−0.003 to 0.012)
High quality	0.001 (−0.001 to 0.004)	−0.011 (−0.015 to −0.006)	−0.003 (−0.011 to 0.005)
Hospice visits when death is imminent			
Low quality	−0.000 (−0.002 to 0.002)	0.010 (0.006 to 0.014)	0.003 (−0.004 to 0.009)
High quality	−0.001 (−0.003 to 0.001)	−0.005 (−0.008 to −0.002)	0.001 (−0.003 to 0.006)
Hospice visits in the last days of life			
Low quality	−0.002 (−0.003 to −0.000)	0.007 (0.003 to 0.010)	0.000 (−0.005 to 0.006)
High quality	−0.004 (−0.006 to −0.002)	−0.009 (−0.013 to −0.005)	−0.001 (−0.007 to 0.006)
HCI: gaps in skilled nursing visits			
Low quality	−0.004 (−0.006 to −0.001)	0.007 (0.002 to 0.012)	0.006 (−0.002 to 0.014)
High quality	−0.001 (−0.003 to 0.001)	−0.005 (−0.008 to −0.002)	−0.003 (−0.008 to 0.001)
HCI: late live discharges			
Low quality	−0.004 (−0.006 to −0.002)	0.003 (−0.001 to 0.008)	0.000 (−0.007 to 0.008)
High quality	0.006 (0.005 to 0.008)	−0.002 (−0.005 to 0.000)	−0.001 (−0.006 to 0.004)
HCI: burdensome transitions, type 1			
Low quality	−0.002 (−0.004 to −0.001)	−0.003 (−0.007 to −0.000)	−0.003 (−0.010 to 0.005)
High quality	0.002 (0.000 to 0.003)	0.002 (−0.002 to 0.005)	−0.001 (−0.005 to 0.003)
HCI: skilled nursing care minutes per routine home care day			
Low quality	−0.001 (−0.003 to 0.000)	0.007 (0.003 to 0.012)	−0.001 (−0.007 to 0.006)
High quality	0.001 (−0.001 to 0.004)	−0.004 (−0.009 to −0.000)	−0.005 (−0.013 to 0.003)
CAHPS global rating			
Low quality	−0.003 (−0.006 to −0.000)	0.028 (0.023 to 0.033)	0.019 (0.009 to 0.029)
High quality	0.001 (−0.001 to 0.003)	−0.016 (−0.020 to −0.013)	−0.014 (−0.022 to −0.006)
Hospice star rating			
Low quality	−0.001 (−0.004 to 0.001)	0.016 (0.011 to 0.021)	0.004 (−0.006 to 0.013)
High quality	0.001 (−0.001 to 0.003)	−0.012 (−0.016 to −0.007)	−0.006 (−0.013 to 0.001)

Abbreviations: CAHPS, Consumer Assessment of Healthcare Providers and Systems; HCI, Hospice Care Index; HIS, Hospice Item Set.

^a^ Beneficiaries with Medicare fee-for-service is the reference for these comparisons.

In sensitivity analyses, our results were robust across models with county fixed effects, models in which the sample was further limited to zip codes with at least 1 high-quality hospice agency, and models in which beneficiaries under 65 years of age were excluded. Differences between MA SNP plan types were small when examining probability of hospice use in the last 6 months of life (eTable 4 in Supplement 1). However, there was heterogeneity when examining the quality of hospices used, with beneficiaries enrolled in I-SNPs showing the largest differences with FFS beneficiaries (eTable 5 in Supplement 1). Differences between MA plan quality tiers (eTables 6 and 7 in Supplement 1) were generally small and inconsistent.

## Discussion

In this cross-sectional study, we found that beneficiaries enrolled in every type of MA plan were significantly more likely than beneficiaries enrolled in FFS to utilize hospice care in the last 6 months of life. Regular MA and FFS beneficiaries used hospices of similar quality. However, beneficiaries in MA SNPs and MMPs were significantly more likely than FFS beneficiaries to use hospices of inferior quality. When beneficiaries were referred by the same hospital or nursing home, the results were attenuated, suggesting upstream referral networks were an important mechanism of the hospice quality choice.

Unlike prior studies on the quality of hospitals, nursing facilities, and home health agencies that are used by FFS and MA beneficiaries,^10,11,12^ we did not find meaningful differences in the quality of hospices used by FFS and regular MA beneficiaries. This is likely a result of MA plans having no financial incentive to steer beneficiaries toward specific hospice providers. Prior studies did not include or did not look separately at beneficiaries in MA SNPs and MMPs, overlooking a key finding of our study.

Our finding that beneficiaries enrolled in MA SNPs and MMPs experienced the largest disparities with FFS beneficiaries in hospice quality is concerning. Although MMPs are being phased out, enrollment in SNPs is growing rapidly, increasing by 24% in the past year alone,^1^ and these plans serve the most vulnerable populations in terms of health, socioeconomic status, and race and ethnicity. Studies indicate that individuals with lower socioeconomic status and individuals from minoritized racial and ethnic groups are more likely to experience poorer end of life care and outcomes, with higher rates of aggressive interventions and care utilization and poorer perceptions of care quality.^19,20,21^ Use of lower-quality hospices may perpetuate and exacerbate existing disparities in quality of care and outcomes.

Our findings suggest some possible mechanisms for observed disparities in the quality of hospices used by FFS and MA beneficiaries. Differences between our unadjusted results and results accounting for zip codes suggest that some disparities are due to geographic availability of high-quality hospices. This may be particularly salient for SNP and MMP beneficiaries, given the limited number of states that offer MMPs and the uneven distribution of SNPs across states and counties.^22^ Additionally, our findings with hospital or nursing home fixed effects support a hypothesis that some of the observed differences in hospice quality may be created by the narrow MA hospital and nursing facility networks. When MA and FFS beneficiaries enter hospice from the same hospital or nursing home, the hospices are more similar in quality, because the referring institution does not differentiate between beneficiaries in different Medicare coverage groups when making the referral.

### Limitations

Our study has several limitations. Not all of the HQRP measures used correspond to the same period of care delivery. The beginning of the Hospice Benefit Component of the Value-Based Insurance Design model overlapped with a portion of the care delivery period of some of our HQRP measures and thus may have influenced these outcomes. A substantial number of hospices were not given a quality rating in the HQRP data files for 1 or more of our outcome measures. Finally, our findings may not be generalizable to beneficiaries residing in areas with extremely low or high MA penetration rates and beneficiaries in rural areas.

## Conclusions

This cross-sectional study provides novel information on the association between MA enrollment and hospice care quality. Regular Medicare Advantage and fee-for-service beneficiaries enrolled in hospices of similar quality. However, beneficiaries in Medicare Advantage special needs plans were significantly more likely than fee-for-service beneficiaries to use hospices of inferior quality, with referral networks playing an important role in hospice quality choice. Given the growth in both MA and hospice enrollment, it is vital that we understand the quality of hospices serving MA enrollees and the mechanisms that underlie the hospice quality choice. As policymakers test new strategies for promoting high-value health care within the MA program, they should consider policies that incentivize referrals to high-quality hospices and approaches to educating beneficiaries on identifying high-quality hospice care.

## Notes

Supplement 1. **eFigure.** Study population flow chart **eTable 1.** Description of Hospice Quality Reporting Program (HQRP) measures **eTable 2.** Distributions of non-Consumer Assessment of Healthcare Providers and Systems (CAHPS) quality measures **eTable 3.** Characteristics of hospice enrollees and the hospices they used by insurance type **eTable 4.** Association between Special Needs Plan (SNP) type and probability of hospice use in the last six months of life **eTable 5.** Association between Special Needs Plan (SNP) type and probability of enrollment in low- and high-quality hospices **eTable 6.** Association between insurance type/quality and probability of hospice use in the last six months of life **eTable 7.** Association between insurance type/quality and probability of enrollment in low- and high-quality hospices

Supplement 2.
Data Sharing Statement
