# Supplementary material for: Quality of Hospices Used by Medicare Advantage and Traditional Fee-for-Service Beneficiaries
Source: JAMA Netw Open. 2024 Dec 16;7(12):e2451227. doi: 10.1001/jamanetworkopen.2024.51227 (PMC11650393; doi:10.1001/jamanetworkopen.2024.51227)
Supplement: Supplement 1. — eFigure. Study population flow chart eTable 1. Description of Hospice Quality Reporting Program (HQRP) measures eTable 2. Distributions of non-Consumer Assessment of Healthcare Providers and Systems (CAHPS) quality measures eTable 3. Characteristics of hospice enrollees and the hospices they used by insurance type eTable 4. Association between Special Needs Plan (SNP) type and probability of hospice use in the last six months of life eTable 5. Association between Special Needs Plan (SNP) type and probability of enrollment in low- and high-quality hospices eTable 6. Association between insurance type/quality and probability of hospice use in the last six months of life eTable 7. Association between insurance type/quality and probability of enrollment in low- and high-quality hospices [file jamanetwopen-e2451227-s001.pdf]

Supplementary Online Content

White LLY, Sun C, Coe NB. Quality of hospices used by Medicare Advantage and traditional fee-for-service beneficiaries. *JAMA Netw Open*. 2024;7(12):e2451227. doi:10.1001/jamanetworkopen.2024.51227

eFigure. Study population flow chart

eTable 1. Description of Hospice Quality Reporting Program (HQRP) measures

eTable 2. Distributions of non-Consumer Assessment of Healthcare Providers and Systems (CAHPS) quality measures

eTable 3. Characteristics of hospice enrollees and the hospices they used by insurance type

eTable 4. Association between Special Needs Plan (SNP) type and probability of hospice use in the last six months of life

eTable 5. Association between Special Needs Plan (SNP) type and probability of enrollment in low- and high-quality hospices

eTable 6. Association between insurance type/quality and probability of hospice use in the last six months of life

eTable 7. Association between insurance type/quality and probability of enrollment in low- and high-quality hospices

This supplementary material has been provided by the authors to give readers additional information about their work.

© 2024 White LLY et al. *JAMA Network Open*.

eFigure. Study population flow chart

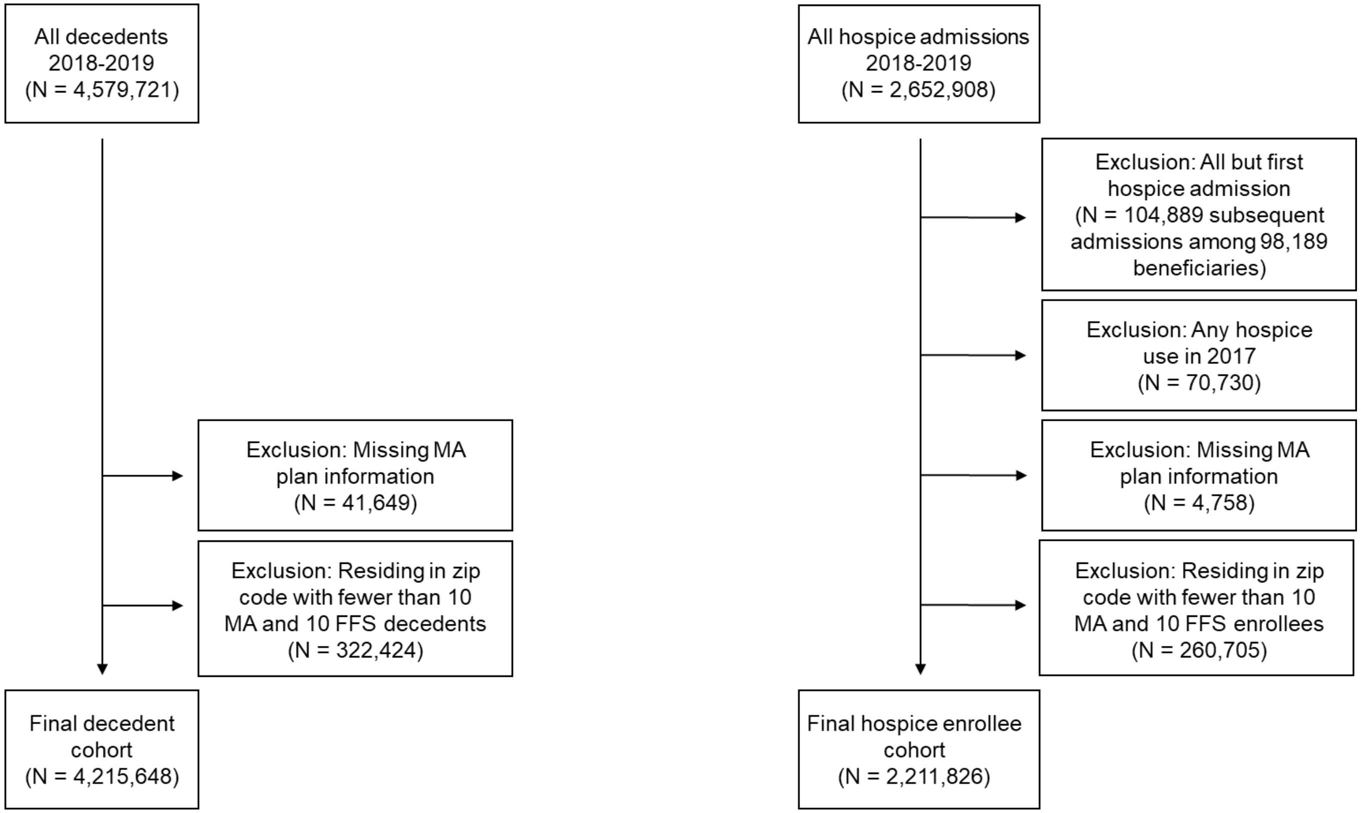

© 2024 White LLY et al. *JAMA Network Open*.

eTable 1. Description of Hospice Quality Reporting Program (HQRP) measures

Measure	Data Source	Score Interpretation	Definition of High/ Low Quality
Hospice Item Set Composite – Comprehensive Assessment at Admission	Hospice-reported	The percentage of patients for whom the hospice performed all 7 care processes, as applicable. The 7 processes include:<br>- Beliefs/values addressed<br>- Treatment preferences<br>- Pain screening<br>- Pain assessment<br>- Dyspnea treatment<br>- Dyspnea screening<br>- Patients treated with an opioid who are given a bowel regimen	Highest/lowest performance quintile
Hospice Visits When Death is Imminent	Hospice-reported	The percentage of patients who received at least 1 visit from a registered nurse, physician, nurse practitioner, or physician assistant in the last 3 days of life.	Highest/lowest performance quintile
Hospice Visits in the Last Days of Life[a]	Claims-based	The proportion of patients who have received in-person visits from a registered nurse or a medical social worker on at least 2 out of the final 3 days of life.	Highest/lowest performance quintile
Hospice Care Index – Gaps in Skilled Nursing Visits	Claims-based	The percentage of hospice stays, of at least 30 days, where the patient experienced at least 1 gap between nursing visits exceeding 7 days.	Highest/lowest performance quintile
Hospice Care Index – Late Live Discharges	Claims-based	The percentage of all live discharges from hospice occurring on or after 180 days after hospice admission.	Highest/lowest performance quintile
Hospice Care Index – Burdensome Transitions, Type 1	Claims-based	The percentage of all live discharges from hospice that were followed by hospitalization within 2 days and followed by hospital readmission within 2 days of hospital discharge.	Highest/lowest performance quintile
Hospice Care Index – Skilled Nursing Care Minutes per Routine Home Care Day	Claims-based	Average total skilled nurse minutes provided by hospices on all Routine Home Care (RHC) service days: the total number of skilled nurse minutes provided by the hospice on all RHC service days divided by the total number of RHC days the hospice serviced.	Highest/lowest performance quintile
CAHPS Global Rating	Survey	Percentage of respondents rating the hospice agency a 9 or 10, where 0 is the worst hospice care possible and 10 is the best hospice care possible.	3 points above/below the national average
Hospice Star Rating	Survey	A summary star rating for the 8 reported CAHPS measures.	High – 3/4 stars<br>Low – 1/2 stars

a – Hospice Visits in the Last Days of Life replaced Hospice Visits When Death is Imminent in 2022

eTable 2. Distributions of non-Consumer Assessment of Healthcare Providers and Systems (CAHPS) quality measures

Quality Measure	N	20th Percentile	80th Percentile	Minimum	Maximum	Median	IQR[a]
HIS[b] Composite	4,081	82.9	98.4	2.7	100	93.8	85.6-97.8
Hospice visits when death is imminent	3,618	78.6	96.1	0	100	90.2	81.6-95.2
Hospice visits in the last days of life	4,667	28.3	71.4	0	100	54.7	34.4-68.8
HCI[c] – Gaps in skilled nursing visits[d]	4,706	32.0	70.7	0	100	52.3	35.6-67.3
HCI – Late live discharges[d]	4,704	22.7	45.3	0	100	33.3	25.0-42.9
HCI – Burdensome transitions, type 1[d]	4,704	1.4	13.6	0	60	7.0	2.6-12.3
HCI – Skilled nursing care minutes per routine home care day	4,706	10.2	16.4	0	338.2	13	10.7-15.7

a – IQR – Interquartile range
b – HIS – Hospice Item Set
c – HCI – Hospice Care Index
d – Measure is reverse-scored

© 2024 White LLY et al. *JAMA Network Open*.

eTable 3. Characteristics of hospice enrollees and the hospices they used by insurance type

		Medicare Advantage (n=844,881)		
	**Traditional Fee-for-Service Medicare**	**Regular Medicare Advantage**	**Special Needs Plan**	**Medicare-Medicaid Plan**
Beneficiary Characteristics				
Beneficiaries, No.[a]	1,366,945	704,585	120,374	19,922
Age, mean (SD)[b], years	82.5 (10.7)	82.7 (9.6)	79.7 (11.4)	80.0 (12.3)
Sex, No. (%)				
Female	777,411 (56.9)	385,642 (54.7)	75,693 (62.9)	12,999 (65.3)
Male	589,534 (43.1)	318,943 (45.3)	44,681 (37.1)	6,923 (34.8)
Race/ethnicity, No. (%)				
African American/Black	104,767 (7.7)	58,558 (8.3)	22,802 (18.9)	3,972 (19.9)
Hispanic/Latinx	64,975 (4.8)	48,587 (6.9)	21,243 (17.7)	3,840 (19.3)
White	1,153,752 (84.4)	576,299 (81.8)	70,113 (58.3)	10,797 (54.2)
Other[c]	36,638 (2.7)	18,514 (2.6)	5,801 (4.8)	1,230 (6.2)
Unknown	6,813 (0.5)	2,627 (0.4)	415 (0.3)	83 (0.4)
Dual enrollment, No. (%)				
Non-dual	1,010,696 (73.9)	603,139 (85.6)	18,222 (15.1)	101 (0.5)
Partial dual	45,860 (3.4)	30,371 (4.3)	18,409 (15.3)	49 (0.3)
Full dual	310,389 (22.7)	71,075 (10.1)	83,743 (69.6)	19,772 (99.3)
Original reason for Medicare entitlement – Disability/ESRD[d], No. (%)	224,411 (16.4)	102,291 (14.5)	36,101 (30.0)	5,641 (28.3)
Hospice diagnosis, No. (%)				
Cancer	361,559 (26.5)	201,105 (28.5)	27,505 (22.9)	3,914 (19.7)
Congestive heart failure	162,734 (11.9)	86,497 (12.3)	13,994 (11.6)	1,999 (10.0)
Chronic obstructive pulmonary disease	85,976 (6.3)	45,384 (6.4)	10,089 (8.4)	1,441 (7.2)
Cerebrovascular accident	48,664 (3.6)	24,729 (3.5)	4,355 (3.6)	837 (4.2)
Dementia	225,280 (16.5)	106,433 (15.1)	20,667 (17.2)	4,141 (20.8)
ESRD	29,984 (2.2)	12,844 (1.8)	2,701 (2.2)	571 (2.9)
All other diagnoses	452,748 (33.1)	227,593 (32.3)	41,063 (34.1)	7,019 (35.2)
Hospitalization within 7 days of hospice admission, No. (%)	608,604 (44.5)	277,009 (39.3)	46,549 (38.7)	7,556 (37.9)
Nursing home use within 7 days of hospice admission, No. (%)	415,114 (30.4)	156,506 (22.2)	48,254 (40.1)	10,949 (55.0)

© 2024 White LLY et al. *JAMA Network Open*.

eTable 3. Characteristics of hospice enrollees and the hospices they used by insurance type (continued)

	Traditional Fee-for-Service Medicare	Medicare Advantage (n=844,881)		
		Regular Medicare Advantage	Special Needs Plan	Medicare-Medicaid Plan
Hospice Characteristics				
Hospice type, No. (%)				
Freestanding	1,147,661 (84.0)	588,308 (83.5)	104,293 (86.7)	17,923 (90.0)
Hospital-based	92,042 (6.7)	48,597 (6.9)	5,974 (5.0)	1,150 (5.8)
Skilled nursing facility-based	1,223 (0.1)	313 (0.0)	129 (0.1)	86 (0.4)
Home health agency	125,913 (9.2)	67,278 (9.6)	9,957 (8.3)	761 (3.8)
Ownership type, No. (%)				
Non-profit	567,841 (41.5)	294,751 (41.8)	41,133 (34.2)	5,895 (29.6)
For profit	611,639 (44.8)	325,100 (46.2)	61,768 (51.3)	12,165 (61.1)
Government owned	20,351 (1.5)	7,914 (1.1)	1,127 (0.9)	278 (1.4)
Other	167,008 (12.2)	76,731 (10.9)	16,325 (13.6)	1,582 (7.9)

a – No. – Number

b – SD – Standard deviation

c – Other race/ethnicity includes the following categories from the Research Triangle Institute (RTI) race variable: Asian/Pacific Islander, American Indian/Alaska Native, and Other

d – ESRD – End-stage renal disease

© 2024 White LLY et al. *JAMA Network Open*.

eTable 4. Association between Special Needs Plan (SNP) type and probability of hospice use in the last six months of life

	Fee-for-Service	Medicare Advantage Special Needs Plan (SNP)		
		**Chronic Condition SNP**	**Dual-Eligible SNP**	**Institutional SNP**
		Coefficient (95% CI)[a]		
Hospice use in the last 6 months of life	Reference group	0.023 (0.017, 0.029)	0.027 (0.023, 0.030)	0.016 (0.008, 0.025)

a – CI – Confidence interval

© 2024 White LLY et al. *JAMA Network Open*.

eTable 5. Association between Special Needs Plan (SNP) type and probability of enrollment in low- and high-quality hospices

Quality Measure	Fee-for-Service	Medicare Advantage Special Needs Plan (SNP)		
		Chronic Condition SNP	Dual-Eligible SNP	Institutional SNP
		Coefficient (95% CI)[a]		
HIS[b] composite				
Low quality	Reference group	-0.003 (-0.008, 0.002)	-0.001 (-0.004, 0.001)	-0.002 (-0.009, 0.005)
High quality		-0.009 (-0.015, -0.003)	-0.008 (-0.012, -0.004)	-0.039 (-0.048, -0.030)
Hospice visits when death is imminent				
Low quality	Reference group	0.004 (-0.002, 0.009)	0.006 (0.002, 0.009)	0.022 (0.013, 0.031)
High quality		-0.002 (-0.007, 0.003)	-0.001 (-0.003, 0.002)	-0.019 (-0.024, -0.013)
Hospice visits in the last days of life				
Low quality	Reference group	0.005 (0.000, 0.009)	0.003 (0.001, 0.005)	0.017 (0.011, 0.023)
High quality		-0.003 (-0.009, 0.002)	0.000 (-0.003, 0.003)	-0.012 (-0.020, -0.004)
HCI[d] – Gaps in skilled nursing visits				
Low quality	Reference group	0.020 (0.014, 0.027)	0.001 (-0.002, 0.004)	0.029 (0.020, 0.038)
High quality		-0.014 (-0.019, -0.009)	0.000 (-0.003, 0.003)	-0.017 (-0.023, -0.011)
HCI – Late live discharges				
Low quality	Reference group	0.010 (0.005, 0.015)	-0.006 (-0.009, -0.004)	0.035 (0.027, 0.043)
High quality		-0.007 (-0.011, -0.003)	0.011 (0.008, 0.014)	-0.024 (-0.030, -0.019)
HCI – Burdensome transitions, type 1				
Low quality	Reference group	0.008 (0.002, 0.014)	-0.005 (-0.007, -0.002)	-0.004 (-0.010, 0.002)
High quality		-0.005 (-0.008, -0.002)	0.005 (0.002, 0.007)	0.006 (-0.001, 0.013)
HCI – Skilled nursing care minutes per routine home care day				
Low quality	Reference group	0.005 (-0.001, 0.011)	-0.000 (-0.003, 0.003)	0.030 (0.021, 0.038)
High quality		-0.023 (-0.031, -0.018)	0.011 (0.008, 0.014)	-0.039 (-0.048, -0.030)
CAHPS[e] global rating				
Low quality	Reference group	0.003 (-0.004, 0.010)	0.019 (0.016, 0.023)	0.127 (0.118, 0.136)
High quality		-0.017 (-0.023, -0.010)	-0.022 (-0.025, -0.019)	-0.079 (-0.087, -0.072)
Hospice star rating				
Low quality	Reference group	0.009 (0.002, 0.016)	0.014 (0.010, 0.017)	0.071 (0.061, 0.080)
High quality		-0.003 (-0.010, 0.005)	-0.016 (-0.019, -0.013)	-0.048 (-0.056, -0.039)

© 2024 White LLY et al. *JAMA Network Open*.

eTable 6. Association between insurance type/quality and probability of hospice use in the last six months of life

	Fee-for-Service	Medicare Advantage				
		Regular Medicare Advantage Lower Quality	Regular Medicare Advantage Higher Quality	Special Needs Plan Lower Quality	Special Needs Plan Higher Quality	Medicare-Medicaid Plan
		Coefficient (95% CI)[a]				
Hospice use in the last 6 months of life	Ref[b]	0.028 (0.026, 0.030)	0.036 (0.035, 0.038)	0.010 (0.006, 0.014)	0.035 (0.031, 0.039)	0.036 (0.030, 0.042)

a – CI – Confidence interval
b – Ref – Reference group

© 2024 White LLY et al. *JAMA Network Open*.

eTable 7. Association between insurance type/quality and probability of enrollment in low- and high-quality hospices

Quality Measure	Fee-for-Service	Medicare Advantage				
		Regular Medicare Advantage Lower Quality	Regular Medicare Advantage Higher Quality	Special Needs Plan Lower Quality	Special Needs Plan Higher Quality	Medicare-Medicaid Plan
		Coefficient (95% CI)[a]				
HIS[b] composite						
Low quality	Ref[c]	0.001 (-0.001, 0.003)	0.002 (0.001, 0.003)	-0.001 (-0.004, 0.003)	-0.002 (-0.005, 0.002)	0.016 (0.010, 0.021)
High quality		0.007 (0.004, 0.010)	-0.005 (-0.006, -0.003)	-0.008 (-0.013, -0.003)	-0.021 (-0.026, -0.017)	-0.010 (-0.018, -0.003)
Hospice visits when death is imminent						
Low quality	Ref	0.004 (0.002, 0.006)	-0.001 (-0.002, 0.001)	0.012 (0.008, 0.016)	0.010 (0.006, 0.014)	0.015 (0.009, 0.020)
High quality		0.003 (0.001, 0.006)	-0.004 (-0.005, -0.003)	-0.002 (-0.006, 0.001)	-0.008 (-0.011, -0.006)	0.001 (-0.003, 0.006)
Hospice visits in the last days of life						
Low quality	Ref	0.004 (0.002, 0.005)	0.001 (-0.000, 0.001)	0.010 (0.007, 0.013)	0.006 (0.004, 0.009)	0.018 (0.013, 0.023)
High quality		-0.008 (-0.010, -0.006)	-0.008 (-0.009, -0.007)	-0.008 (-0.012, -0.004)	-0.002 (-0.006, 0.002)	-0.001 (-0.006, 0.005)
HCI[d] – Gaps in skilled nursing visits						
Low quality	Ref	-0.005 (-0.007, -0.002)	-0.000 (-0.002, 0.001)	0.003 (-0.002, 0.007)	0.018 (0.015, 0.022)	0.027 (0.020, 0.034)
High quality		-0.004 (-0.005, -0.002)	-0.002 (-0.003, -0.001)	-0.007 (-0.011, -0.004)	-0.009 (-0.012, -0.006)	-0.006 (-0.010, -0.002)
HCI – Late live discharges						
Low quality	Ref	-0.002 (-0.003, 0.000)	-0.004 (-0.005, -0.003)	-0.005 (-0.009, -0.001)	0.013 (0.009, 0.017)	-0.001 (-0.007, 0.005)
High quality		-0.000 (-0.002, 0.002)	0.012 (0.011, 0.014)	0.002 (-0.001, 0.006)	-0.002 (-0.004, 0.001)	-0.006 (-0.011, -0.001)
HCI – Burdensome transitions, type 1						
Low quality	Ref	-0.003 (-0.005, -0.001)	-0.003 (-0.004, -0.002)	-0.004 (-0.007, -0.001)	-0.004 (-0.007, -0.001)	0.020 (0.014, 0.027)
High quality		-0.002 (-0.003, -0.000)	0.018 (0.016, 0.019)	-0.006 (-0.009, -0.003)	0.009 (0.005, 0.012)	-0.002 (-0.006, 0.002)
HCI – Skilled nursing care minutes per routine home care day						
Low quality	Ref	0.004 (0.002, 0.006)	-0.003 (-0.004, -0.001)	0.015 (0.010, 0.019)	0.008 (0.004, 0.012)	0.000 (-0.006, 0.007)
High quality		-0.009 (-0.011, -0.007)	0.004 (0.002, 0.005)	-0.002 (-0.006, 0.001)	-0.011 (-0.016, -0.007)	-0.006 (-0.013, 0.001)
CAHPS[e] global rating						
Low quality	Ref	0.003 (0.000, 0.006)	0.002 (0.000, 0.004)	0.036 (0.031, 0.041)	0.048 (0.044, 0.053)	0.068 (0.060, 0.077)
High quality		-0.006 (-0.008, -0.004)	-0.004 (-0.006, -0.003)	-0.027 (-0.031, -0.022)	-0.037 (-0.041, -0.033)	-0.047 (-0.054, -0.040)
Hospice star rating						
Low quality	Ref	0.001 (-0.001, 0.004)	0.002 (0.000, 0.003)	0.026 (0.021, 0.031)	0.030 (0.025, 0.034)	0.028 (0.020, 0.036)
High quality		0.005 (0.002, 0.009)	-0.005 (-0.006, -0.003)	-0.019 (-0.024, -0.014)	-0.024 (-0.028, -0.020)	-0.025 (-0.032, -0.019)

a – CI – Confidence interval
b – HIS – Hospice Item Set
c – Ref – Reference group
d – HCI – Hospice Care Index
e – CAHPS – Consumer Assessment of Healthcare Providers and Systems

© 2024 White LLY et al. *JAMA Network Open*.
