# Supplementary material for: Quality of Hospices Used by Medicare Advantage and Traditional Fee-for-Service Beneficiaries
Source: JAMA Netw Open. 2024 Dec 16;7(12):e2451227. doi: 10.1001/jamanetworkopen.2024.51227 (PMC11650393; doi:10.1001/jamanetworkopen.2024.51227)
Supplement: Supplement 2. — Data Sharing Statement [file jamanetwopen-e2451227-s002.pdf]

Data Sharing Statement

White. Quality of Hospices Used by Medicare Advantage and Traditional Fee-for-Service Beneficiaries. *JAMA Netw Open*. Published December 16, 2024.
doi:10.1001/jamanetworkopen.2024.51227

Data
Data available: No
